# Supplementary material for: The hydrolase LpqI primes mycobacterial peptidoglycan recycling
Source: Nat Commun. 2019 Jun 14;10:2647. doi: 10.1038/s41467-019-10586-2 (PMC6572805; doi:10.1038/s41467-019-10586-2)
Supplement: Supplementary file 1 — Supplementary Information [file 41467_2019_10586_MOESM1_ESM.pdf]

**Supplementary Information for:**

# The hydrolase LpqI primes mycobacterial peptidoglycan recycling

Patrick J. Moynihan[1]*, Ian T. Cadby[1], Natacha Veerapen[1], Monika Jankute[1], Marialuisa Crosatti[2], Galina V. Mukamolova[2], Andrew L. Lovering[1] and Gurdyal S. Besra[1]

**Affiliations:**
[1]Institute of Microbiology and Infection
School of Biological Sciences
University of Birmingham
Birmingham, UK, B15 2TT

[2]Leicester Tuberculosis Research group, Department of Respiratory Sciences, University of Leicester, Leicester, LE1 9HN, UK

*To whom correspondence should be addressed:
Patrick J. Moynihan, Ph.D.,
Institute of Microbiology and Infection
School of Biological Sciences
University of Birmingham
Birmingham, UK, B15 2TT
email: p.j.moynihan@bham.ac.uk

**Supplementary Table 1. Primers used in this study.**

| Primer name | Primer sequences (5'-3') |
|---|---|
| RR-Rv0237 | TTTTTTTTCCATAGATTGGCGTCAGCGAGGCGGTGTT |
| RL-Rv0237 | TTTTTTTTCCATCTTTTGGCAACGTCGGTGTCACCGA |
| LR-Rv0237 | TTTTTTTTCCATAAATTGGGGCGCACCGAGCGGCCAG |
| LL-Rv0237 | TTTTTTTTCCATTTCTTGGCGCCGGCTCGGCGCAGCT |
| Rv0237CompF | CTAGGAATTCATCAGGGTCACTCCGAAG |
| Rv0237CompR | CTAGAAGCTTCTAACGGCCACACCCCGG |
| Rv0237-SUMOF | AGCCATGGCGGCACACC |
| Rv0237SUMOR | CTAACGGCCACACCCCGGG |
| RpfBFwd | GCATGCAAAACGGTGACG |
| RpFBRev | TCAGCGCGCACCCGCTCG |

**Supplementary Table 2. Distribution of recycling genes in representative bacteria.**

| | | Gram - | | Gram + | | Actinomycetales | | | | |
|---|---|---|---|---|---|---|---|---|---|---|
| | | *E.c.* | *P.p.* | *S.a.* | *B.s.* | *C.g.* | *M.s.* | *M.t.* | *M.l.* | *M.b.* |
| **PG-Fragment Uptake** | *ampG* | + | + | - | - | - | - | - | - | - |
| | *murP* | + | - | + | + | - | - | - | - | - |
| | *nagE* | + | - | + | + | - | + | - | - | - |
| | *murT* | - | - | - | - | - | - | - | - | - |
| **Amino sugar Recycling** | *amgK* | - | + | - | - | - | - | - | - | - |
| | *anmK* | + | + | - | - | - | + | - | - | - |
| | *mupP* | - | + | - | - | - | - | - | - | - |
| | *murK* | - | - | - | - | - | + | - | - | - |
| | *murQ* | + | - | + | + | - | + | - | - | - |
| | *murU* | - | + | - | - | - | - | - | - | - |
| | *nagA* | + | + | + | + | + | + | + | + | + |
| | *nagB* | + | + | + | + | + | + | - | - | - |
| | *nagC* | + | + | + | + | + | + | + | + | + |
| | *nagK* | + | - | - | - | - | - | - | - | - |
| | *nagZ* | + | + | - | + | + | + | + | + | + |
| **Peptide Recycling** | *aeeP* | + | + | - | + | - | - | - | - | - |
| | *amiD* | + | + | - | - | - | - | - | - | - |
| | *ampD* | + | + | - | + | - | - | - | - | - |
| | *ampR* | - | - | - | - | - | - | - | - | - |
| | *ldcA* | + | + | - | + | - | - | - | - | - |
| | *mpaA* | + | - | + | + | - | - | - | - | - |
| | *mpl* | + | + | - | - | - | - | - | - | - |
| | *mppA** | + | + | + | + | + | + | + | + | + |

The genomes of several representative bacteria were analysed for known PG-recycling genes using BLAST. Orthologs identified at an E-value cut-off $10^{-5}$ or lower were then filtered for previously reported functions. *E. coli* (*E.c.*; taxid: 511145), *Pseudomonas putida* (*P.p.*; taxid: 160488) *Staphylococcus aureus* (*S.a.*; taxid: 93061), *Bacillus subtilis* (*B.s.*; taxid: 224308), *C. glutamicum* (*C.g.*; taxid: 196627), *M. smegmatis* (*M.s.*; taxid: 246196), *M. tuberculosis* H37Rv (*M.t.*; taxid: 83332), *M. leprae* (*M. l.*; taxid: 1769) and *M. bovis* BCG (*M.b.*; taxid: 410289). *Oligopeptide binding proteins are highly conserved, but specific substrate requirements remain unclear for most identified orthologs. Similarly, *nagC* orthologs are hard to predict by sequence alone and so these may not represent true orthologs.

**Supplementary Table 3. Data collection and refinement statistics.**

| **Data collection** | |
|---|---|
| Space group | C2 |
| Cell dimensions | |
| $a$, $b$, $c$ (Å) | 121.8, 89.6, 87.1 |
| $\alpha$, $\beta$, $\gamma$ (°) | 90, 125.1, 90 |
| Resolution (Å) | 1.96 (1.96 – 2.07)* |
| $R_{sym}$ | 12.7 (61.2) |
| $R_{pim}$ | 11.5 (56.1) |
| $I$ / $\sigma I$ | 5.6 (1.4) |
| Completeness (%) | 99.9 (99.9) |
| Redundancy | 3.6 (3) |
| **Refinement** | |
| Resolution (Å) | 1.96 |
| $R_{work}$ / $R_{free}$ | 21.6/26.7 |
| rmsd bonds | 0.008 |
| rmsd angles | 1.17 |
| **PDB ID code 6GFV** | |

*Values in parentheses are for highest-resolution shell.

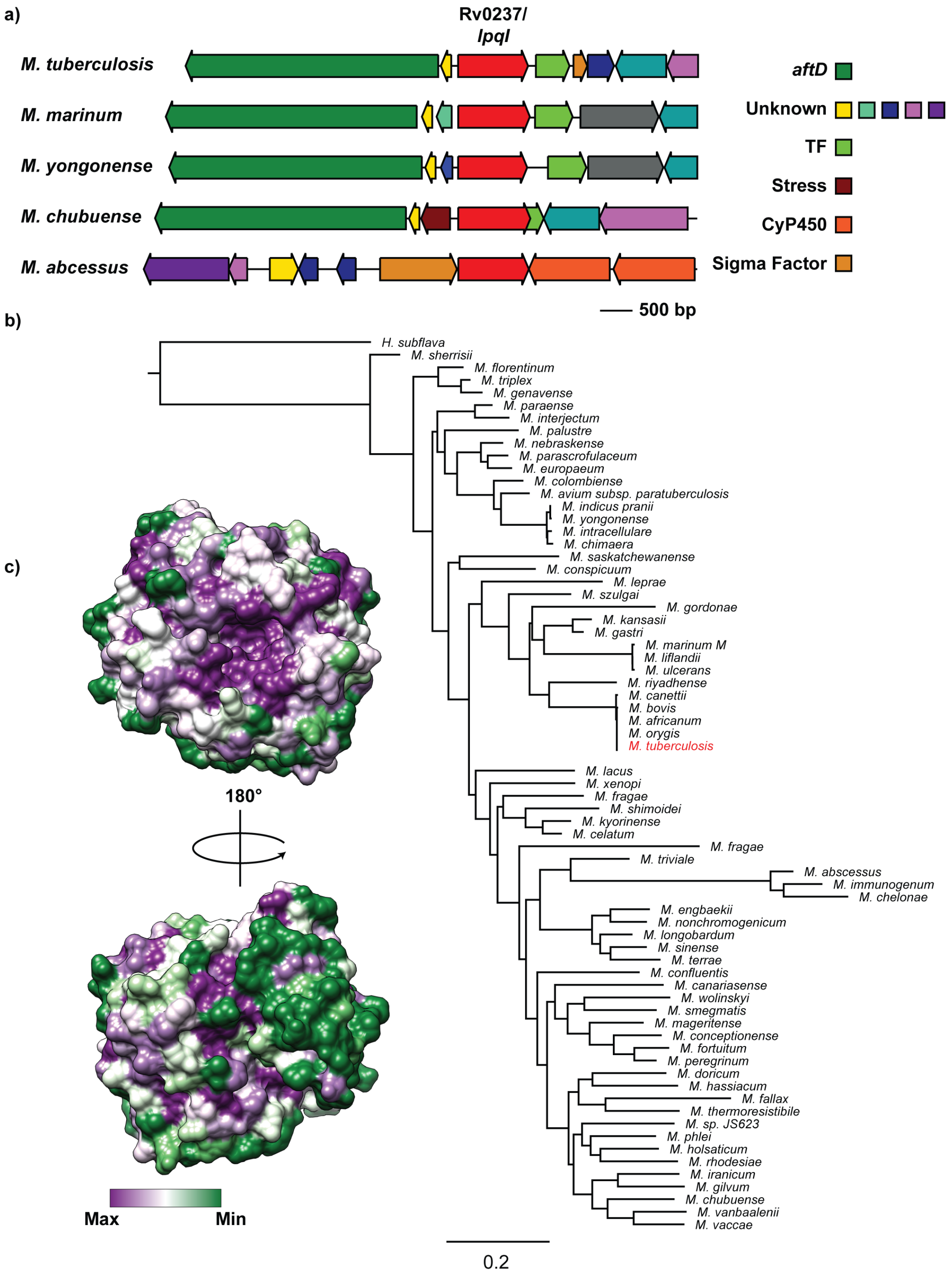


**Supplementary Figure 1. LpqI is conserved in mycobacteria. a)**The PATRIC database was used to view the genomic context of *lpqI* in 50 representative mycobacterial genomes. The gene was largely syntenic with the exception of *lpqI* in *M. abscessus* and closely related species. TF – transcription factor; Stress – universal stress response protein; CyP450 – cytochrome P450. **b)** The coding sequence for *lpqI* was extracted for a representative group of mycobacteria. These were aligned using ClustalW and a tree was constructed using the RAxML ver. 8.2.11 rapid hill-climbing algorithm[1,2]. **c)** The amino-acid sequence for the genes used in b) were aligned using ClustalW and this alignment along with chain A of the LpqI crystal structure were submitted to the ConSurf server[3]. The ConSurf output is colour coded based on conservation of the residues. Notably the active site of the protein is completely conserved (top).

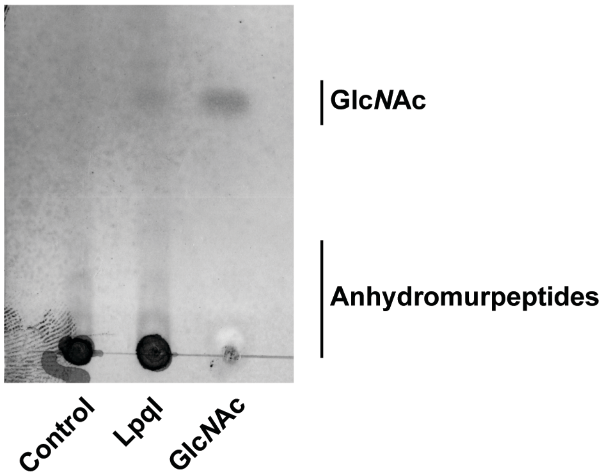


**Supplementary Figure 2. LpqI degrades anhydromuropeptides.** LpqI (1 µM) was incubated with 0.1 mM soluble anhydromuropeptides derived from RpfB-digested *M. smegmatis* mc$^2$155 PG and the reaction products were spotted onto a TLC which was eluted with a mobile phase consisting of 1-butanol, methanol, ammonium hydroxide and water at a ratio of 5:4:2:1 and stained with α-naphthol followed by charring.

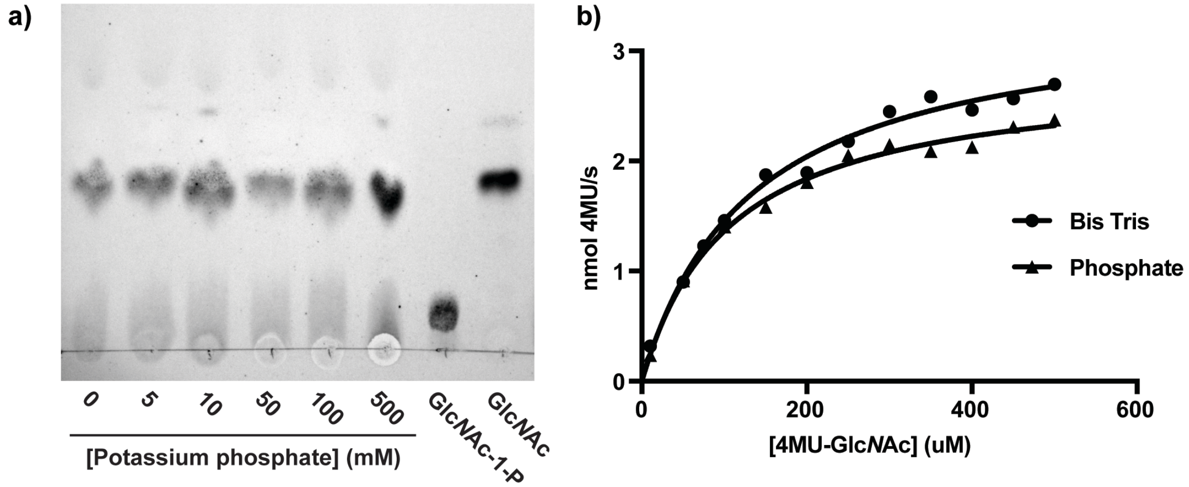


**Supplementary Figure 3. LpqI does not possess phosphorylase activity. a)** Following the protocols in Macdonald *et al.* (2014) [4] LpqI was tested for phosphorylase activity. Reactions containing 5 µM LpqI, 10 mM *p*NP-Glc*N*Ac, 50 mM HEPES (pH 7.0), 300 mM NaCl, 5 mM $MgSO_4$, 1 mM DTT and the indicated concentrations of potassium phosphate were incubated for 15 min at 37 °C. 5 µL of each reaction in addition to standards of Glc*N*Ac-1-P and Glc*N*Ac (10 mM) were spotted on silica TLC plates which were eluted with a mobile phase consisting of 1-butanol, methanol, ammonium hydroxide and water at a ratio of 5:6:4:1 and stained with α-naphthol followed by charring. **b)** In identical buffers to the 500 mM and 0 mM lanes in panel a), LpqI was incubated with increasing concentrations of 4MU-Glc*N*Ac. The rate of 4MU release was plotted and the curve fit with the Michaelis-Menton equation using GraphPad Prism (n = 3; error bars are ± SD).

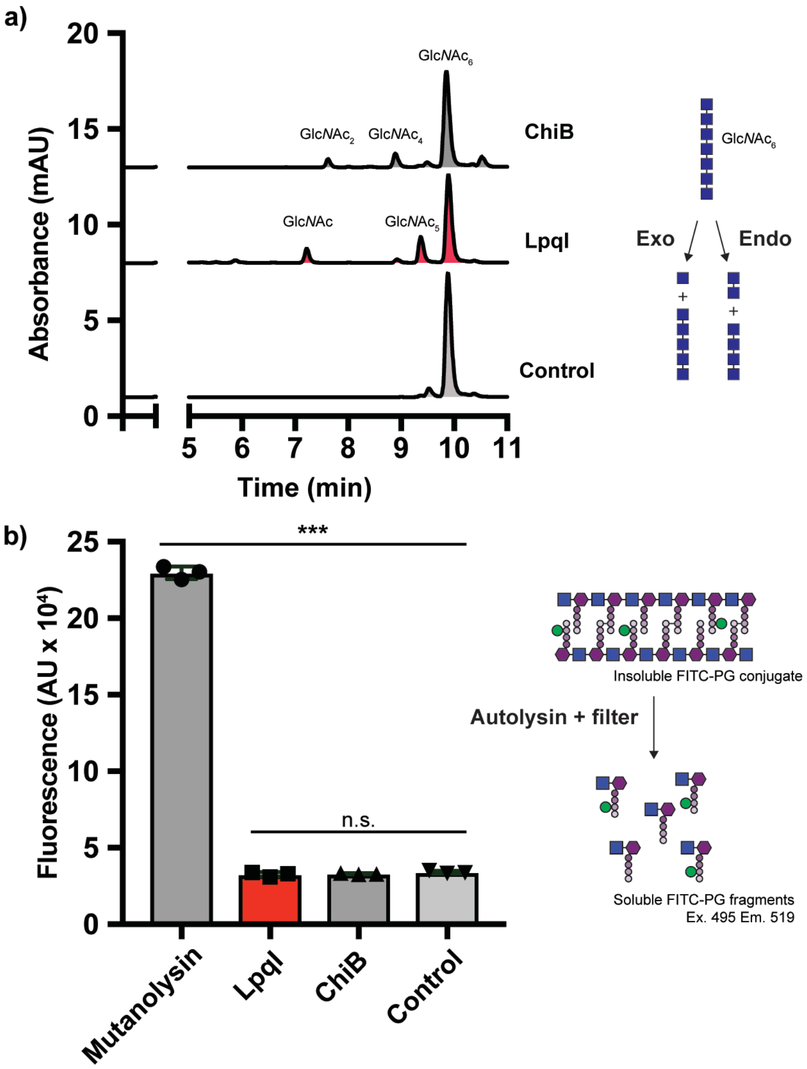


**Supplementary Figure 4. Biochemical characterisation of LpqI.** a) LpqI or ChiB were incubated with Glc*N*Ac$_6$ and the reaction products were analysed by graphitised carbon HPLC. Solvent peaks are omitted from the chromatogram. Representative data from 3 biological replicates is shown. b) LpqI, mutanolysin or ChiB were incubated with FITC-labelled *M. smegmatis* mc$^2$155 PG. The reactions were filtered and the fluorescence of the soluble fraction is reported (n = 3; error bars are ± SD).

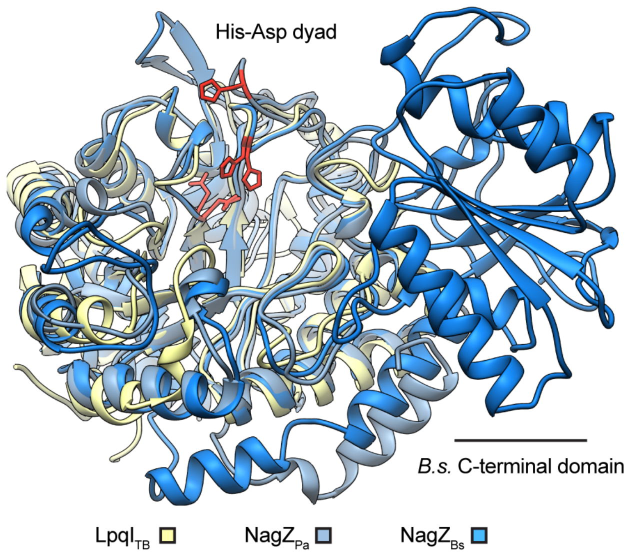


**Supplementary Figure 5. Structural alignment of LpqI with NagZ$_{Pa}$ and NagZ$_{Bs}$.** The SSM Superpose function in Coot was used to align LpqI with the NagZ/Glc*N*Ac/1,6-anhydroMur*N*Ac complex from *Pseudomonas aeruginosa* (NagZ$_{Pa}$; PDB:5G3R) and NagZ from *Bacillus subtilis* (PDB:4GYJ). This yielded an a RMSD of 2.30 Å with 229 residues aligned for NagZ$_{Pa}$ and 1.63 Å with 285 residues aligned for NagZ$_{Bs}$.

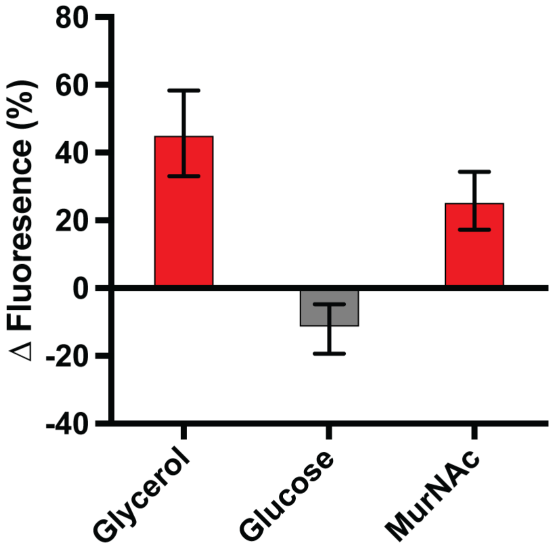


**Supplementary Figure 6. Use of Mur*N*Ac as a sole carbon source is not inhibited by 2-deoxyglucose.** *M. bovis* BCG was cultured in the indicated carbon sources with and without 0.1% 2-deoxyglucose. After one week the growth of the culture was evaluated using a resazurin assay. Reported values are the percent difference of the mean fluorescence of the cultures with and without 2DG added plus or minus one standard deviation (n = 3; error bars are ± SD).

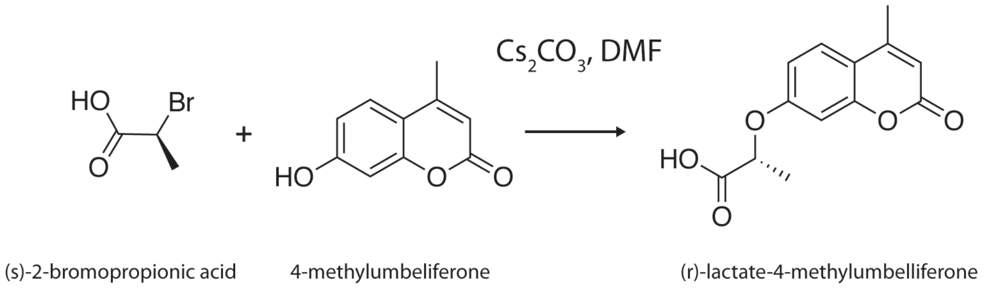


**Supplementary Figure 7. Synthesis of 4MU-D-lactate.** We developed a 1-step synthesis of 4MU-D-lactate. Briefly, 1.5 g of (*s*)-2-bromopropionic acid was added to 1 g of 4-methylumbelliferone stirring in 40 mL dry dimethylformamide and 0.75 g $Cs_2CO_3$.This was stirred at room temperature overnight and the product was extracted three times with water/ethyl-acetate and the organic phase was dried over sodium sulfate. The organic phase was then filtered and evaporated to dryness. The product was subsequently purified using silica chromatography and was dried as a crystalline white solid.

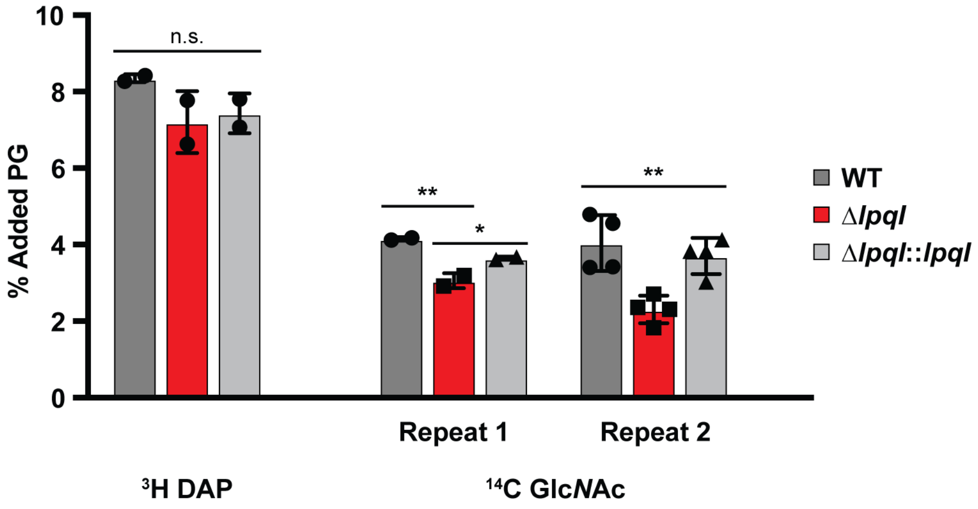


**Supplementary Figure 8. Incorporation of radio-labelled PG by *M. bovis* BCG.** *M. bovis* BCG WT, Δ*lpqI* and Δ*lpqI*::*lpqI* were inoculated at an $OD_{600}$ of 0.01 and incubated with 30,000 CPM of $^{14}$C Glc*N*Ac-labelled muropeptides or 100, 000 CPM of $^{3}$H DAP-labelled muropeptides for 10 days with aeration after which the cell wall material was isolated and subjected to liquid scintillation counting ($^{3}$H DAP n = 2; $^{14}$C Glc*N*Ac Repeat 1 n = 2, Repeat 2 n = 4; ** = $p < 0.005$, * = $p < 0.05$; error bars are ± SD).

## Supplementary References

1. Larkin, M. A. *et al.* Clustal W and Clustal X version 2.0. *Bioinformatics* **23,** 2947–2948 (2007).
2. Stamatakis, A. RAxML-VI-HPC: maximum likelihood-based phylogenetic analyses with thousands of taxa and mixed models. *Bioinformatics* **22,** 2688–2690 (2006).
3. Ashkenazy, H. *et al.* ConSurf 2016: an improved methodology to estimate and visualize evolutionary conservation in macromolecules. *Nucleic Acids Res* **44,** W344–50 (2016).
4. Macdonald, S. S., Blaukopf, M. & Withers, S. G. *N*-Acetylglucosaminidases from CAZy Family GH3 Are Really Glycoside Phosphorylases, Thereby Explaining Their Use of Histidine as an Acid/Base Catalyst in Place of Glutamic Acid. *J Biol Chem* **290,** 4887–4895 (2015).
